# Economic inequalities amongst women with osteoporosis-related fractures: an application of concentration index decomposition

**DOI:** 10.15171/hpp.2016.31

**Published:** 2016-10-01

**Authors:** Rahmatollah Moradzadeh, Haidar Nadrian, Farzaneh Golboni, Mohammad Hasan Kazemi-Galougahi, Nasrin Moghimi

**Affiliations:** ^1^Department of Epidemiology, School of Health, Arak University of Medical Sciences, Arak, Iran; ^2^Department of Health Education & Promotion, Faculty of Health Sciences, Tabriz University of Medical Sciences, Tabriz, Iran; ^3^Department of Midwifery, Faculty of Nursing and Midwifery, Kermanshah University of Medical Sciences, Kermanshah, Iran; ^4^Department of Epidemiology and Biostatistics, School of Health and Health Research Institute, Tehran University of Medical Sciences, Tehran, Iran; ^5^Department of Rheumatology, School of Medicine, Kurdistan University of Medical Sciences, Sanandaj, Iran

**Keywords:** Economic status, Inequalities, Osteoporosis, Bone fracture

## Abstract

**Background:** Considering the renewed emphasis on women’s health, attention to the new aspects of their health, such as equity, among different groups is warranted. The aim of this study was to investigate the economic inequalities among women with osteoporosis-related bone fractures (ORBFs) in Sanandaj, Iran.

**Methods:** In this cross-sectional study, convenient sampling was employed to recruit 220 women with osteoporosis referring to the only rheumatology clinic in Sanandaj (the center of Kurdistan province in Iran) from January to April 2013. Main outcome was the history of fractures due to osteoporosis. Concentration index decomposition (CID) and logistic regression were used for data analysis.

**Results:** In multivariate logistic analysis, the fourth and fifth quintiles of family economic status were found to be significantly associated with ORBFs. Risk difference and confidence interval (CI) for the relation between the history of bone fracture and family economic status was -0.115 (95% CI: -0.209, -0.021; P = 0.016), which reflected the higher prevalence of bone fractures among women with the lower economic levels. About 25% out of all ORBFs were happened among 20% of the women with low economic status.

**Conclusion:** It was concluded that economic status plays an important role in happening ORBFs among underprivileged women. A reorientation on women’s health care services in Iran with a focus on underprivileged postmenopausal women seems to be necessary. There is a need for inter-sectoral coalition between the policymakers of the health system and those of other organizations to reduce the economic inequalities among osteoporotic women.

## Introduction

Osteoporosis is a chronic and progressive disease characterized by decreased bone mass and bone deterioration which may result in bone fracture^1^ and, consequently, decrease in quality of life.^2^

Until 2025, along with the increasing rate of elderly population, it is estimated that the rate of osteoporosis-related fractures to be increased by twice, and consequently, the direct and indirect costs associated with these fractures to be increased, significantly.^3^ It is, also, expected that in the next 50 years, more than75% of osteoporosis-related fractures to be occurred in the developing countries.^4^

In the comprehensive plan for osteoporosis prevention conducted in Iran (2001), it was revealed that 50% of older than 50 men and 70% of their counterpart women, had been diagnosed with osteoporosis or osteopenia.^5^ Derakhshan et al, in a previous study in Kurdistan, reported that the prevalence of osteoporosis among postmenopausal women was 34.4%.^6^ Based on another study conducted in Iran, the average hospitalization costs was US$774 and the average hospitalization days per patient was 9.7 ranging from 5 to 38 days.^7^

Nowadays, beside some indicators showing the average level of population health, there should be taken into account some other factors which may indicate the distribution of diseases among individuals and population groups. Considering the new emphasis on women’s health, as one of the Millennium Development Goals, as well as the significant improvements happened in its central indices in many countries, paying attention to the other new aspects of women’s health, such as social inequalities and equity and their distribution among different population groups is recommended.^8^

The inequality in health has been recognized from centuries ago.^9^ Even in generally wealthy Western countries, material deprivation and poverty are not uncommon.^10^ However, little knowledge exists about economic inequalities in fractures caused by osteoporosis. Moreover, investigating economic inequality among women with specific diseases is a new subject and the number of published studies conducted on such diseases, specifically, osteoporosis in developing countries is scarce. So, it can be claimed that the present study is a novel research on economic inequalities among the women with osteoporosis referring to a rheumatology clinic in Sanandaj, Iran. This rheumatology clinic was situated in one of the central streets of the city and it was assumed to be the only specific clinic for the rheumatic patients which could be available for all the patients with various economic statuses throughout the city. As a new approach in health inequality studies, in the present study, the family income index reported by the female osteoporotic patients was applied as a direct indicator to measure their economic status. Therefore, the aim of this study was to investigate economic inequalities among women with osteoporosis-related fractures in Sanandaj, Iran.

## Material and Methods

### Participants and procedures

**Figure 1:**
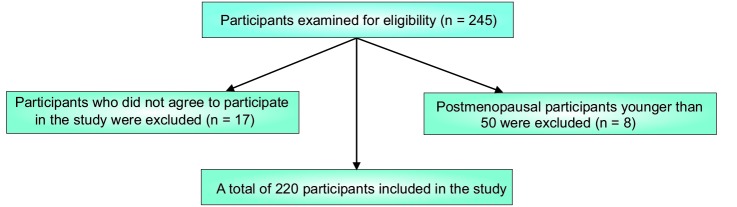
The number of participants included in and/or excluded from the study.

In this cross-sectional study, convenient sampling was employed to recruit 220 women with osteoporosis referring to the only rheumatology clinic in Sanandaj from January to April 2013. Considering the non-probability sampling technique used in this study, during the data collection, all the osteoporotic patients referring to the abovementioned clinic were included in the study. The patients were diagnosed with osteoporosis by a rheumatology specialist physician based on DEXA method and Lunar DPX. Inclusion criteria for the study were postmenopausal women (50-75 years old) with T-score less than or equal to -2.5 which was measured at two points in their hip and spine. Exclusion criteria were postmenopausal women younger than 50 and women who did not agree to participate in the study. Twenty-five respondents did not meet the inclusion criteria and, thus, excluded from the study (Figure 1).

All the respondents were interviewed face to face by an expert nurse in osteoporosis. The interviewer was not aware of the study hypotheses. To reduce the selection bias, all the patients referring to the clinic were included in the study if they have the inclusion criteria. To control the information bias (misclassification in covariates), it was tried to define the variables for participants, clearly. The interviewer was trained on the subject and was asked to apply the same method of data collection for all the participants.

### Measures

A series of questions and tools regarding socio-economic, demographic and anthropometric characteristics of the respondents including age (years), marital status (married/single or divorced/widowed), job (housekeeper/employed/retired), education status (illiterate/elementary/high school/diploma), history of fractures due to osteoporosis (yes/no), weight (kg), height (cm), body mass index (BMI) as well as family income (monthly family income in Rial) was designed. The weight and the height of the respondents were measured applying the digital scale model Beurer BG55 and the Stadiometer model Seca 222, respectively. All the measuring processes of BMI for all the patients were conducted by a rheumatologist in the clinic.

In this study, the self-reported family monthly income was considered as an indicator for the patients’ economic status. As Oakes and Kaufman^11^ suggested, monthly income is one of the main indicators for representing the economic status among populations. Chandola and Marmot^12^ also, recommended income, wealth and assets as good indicators of an individual’s situation in the labor market and his/her material standards of living. Despite these recommendations, there have been reported some limitations for this indicator including the possibility of measurement biases and other possible problems such as measurement error, missing data and non-responsiveness.^12^ In the health economics literature, proxies including consumption and assets indices have been proposed to overcome such possible problems.

In the present study, we chose to use family monthly income as the only indicator for economic status of the respondents due to some limitations reported in “Limitation” section of this paper. However, in order to alleviate the above-mentioned possible problems, it was decided to collect data through interview in a private room. During interviews, the respondents were explained about the purpose of the study, the anonymity and the privacy of the data and, also, the fact that the question on monthly income was only for research objectives.

### Statistical analysis

Firstly, all the subjects were divided into 5 subgroups based on the range of their monthly income, and, secondly, 20% of the first group and 20% of the last group representing the lowest and the highest level of economic status, respectively, were selected.

Concentration index decomposition (CID) is one of the most common methods for measuring inequality. The CID was made based on concentration curve (CC). This index along with its related CC provides a means for measuring the degree of income-related inequality in a specific health variable.

The concentration index (CI) was defined as twice the area between the CC and the line of equality (the 45 degree line running from the bottom-left corner to the top-right). The index takes a negative value when the curve, called as Lorenz curve (LC), lies above the line of equality, indicating disproportionate concentration of the health variable among the poor patients, and a positive value is when it lies below the line of equality. If the health variable is ‘bad,’ such as ill-health, a negative value of the CI means ill-health is higher among the poor patients.^13^

CI was used to analyze the distribution size of income and wealth which measures inequality and poverty among populations.^14^ The Gini coefficient was used to measure inequality in the distribution of income. Decomposing this measure helped us to understand the determinants of inequality.^15^

Logistic regression was used to estimate odds ratios (ORs) and 95% confidence intervals (CIs) for associations between the variables and the history of osteoporosis-related bone fractures (ORBFs) adjusted for other covariates. Data analysis was conducted applying STATA 12.0 software for Windows.

## Results

The total number of osteoporotic women examined for eligibility was 245. Figure 1 illustrates the number of respondents included in or excluded from the study. Eventually, the data obtained from 220 women were analyzed (the response rate = 93.06%).

The socio-economic and underlying characteristics of the osteoporotic women are shown in Table 1. The majority of the respondents were housewife (86.4%), married (90.9%) and illiterate/with elementary education (77.3%). About 40% had a history of ORBF. Also, 18.2% had a BMI more than 25 and about 7% were wealthy.

As there is shown in Table 2, the history of ORBFs among women situated in the second quintile of the economic variable were 2.45 times compared to their counterparts (OR 2.45; 95% CI 0.92 - 6.54) (adjusted OR for covariates). Adjusted ORs between the third, the fourth and the fifth quintiles and ORBFs were 0.70 (95% CI: 0.28-1.74), 0.25 (95% CI: 0.09-0.72) and 0.06 (95% CI: 0.01-0.76), respectively. The OR after adjustment for age was 1.21 and statistically significant (95% CI: 1.12-1.29). In contrast, the OR in patients with a higher BMI (more than 25 kg/m^2^) was 2.59 (95% CI: 0.98-6.89] which was not statistically significant. The results, also, showed that being with a high school/diploma degree [OR: 0.47; 95% CI: 0.12-1.83] and married (OR: 0.24; 95% CI: 0.06-0.89) (adjusted for covariates) decreased the odds of ORBFs among the study participants, although the latter was not statistically significant.

**Table 1:** Socio-economic and underlying characteristics of the respondents

**Variable**	**Mean (SD)**	**No.**	**%**
Age	59.9 (7.01)	220	
Job			
Housekeeper		190	86.4
Employed/retired		30	13.6
Marital status			
Married		200	90.9
Divorced		20	9.1
Education level			
Illiterate /less than high school		170	77.3
High school/diploma		50	22.7
History of fracture			
No		131	59.6
Yes		89	40.5
Weight	68.14 (9.36)		
Height	152.77 (535)		
BMI	22.31 (3.04)		
Less than 25		180	81.8
More than 25		40	18.2
Family monthly income^a^			
The first quintile		45	20.5
The second quintile		48	21.8
The third quintile		62	28.2
The fourth quintile		50	22.7
The fifth quintile		15	6.8
T-score	-3.14 (0.65)		

Abbreviations‏: SD, standard deviation; BMI, body mass index. ^a^To IR Rials.

**Table 2:** Adjusted ORs for covariates associated with ORBF among the participants

**Variables**	**OR (95% CI)**
Job	
Housekeeper	1
Employed/retired	23.26 (3.79, 142.89)
Marital Status	
Married	0.24 (0.06, 0.89)
Divorced	1
Education level	
Illiterate / Less than high school	1
High school/ Diploma	0.47 (0.12, 1.83)
Age	1.21 (1.12, 1.29)
BMI	
Less than 25	1
More than 25	2.59 (0.98, 6.89)
Family Monthly Income	
The first quintile	1
The second quintile	2.45 (0.92, 6.54)
The third quintile	0.70 (0.28, 1.74)
The fourth quintile	0.25 (0.09, 0.72)
The fifth quintile	0.06 (0.01, 0.76)

Abbreviations: OR, odds ratio; BMI, body‏ mass index; ORBF, osteoporosis-related bone fracture‏.

In univariate analysis, the fourth (OR: 0.20 [95% CI: 0.08, 0.52]) and the fifth (OR: 0.07 [95% CI: 0.01, 0.62]) quintiles of economic status were found to be significantly associated with the higher rates of ORBFs. Non-significant positive associations were found between the second (OR: 2.30 [95% CI: 0.99, 5.35]) and the third (OR: 0.71 [95% CI: 0.33, 1.53]) quintiles and ORBFs.

The CI for ORBFs was -0.115 (95% CI: -0.209 and -0.021; *P *= 0.16), which reflects the higher prevalence of bone fractures among women with lower economic levels. Calculated decompositions to obtain the decomposition of explanatory variables for the history of ORBFs are presented in Table 3. The economic status variable in the first quintile explained the highest % age (41.2%) followed by the second quintile within which 28.5% of variation was explained.

**Table 3:** Decomposing socio-economic inequalities among women with the history of osteoporotic fracture in Sanandaj

**Variables**	**Elasticity**	**Concentration Index**	**Contribution**	**Contribution %**
BMI	0.03	-0.06	-0.01	0.7
Age	0.17	-0.05	-0.01	2.7
Marital status	-0.02	0.03	-0.01	0.2
Economic situation in the first quintile	0.17	-0.80	-0.14	41.2
Economic situation in the second quintile	0.25	-0.37	-0.095	28.5
Economic situation in the third quintile	0.22	0.13	0.028	8.6
Economic situation in the fourth quintile	0.09	0.64	0.06	17.5
Economic situation in the fifth quintile	0	0.94	0	0
Education status	0.09	-0.03	-0.01	0.80

‏ Abbreviation‏: BMI, body mass index.

**Figure 2:**
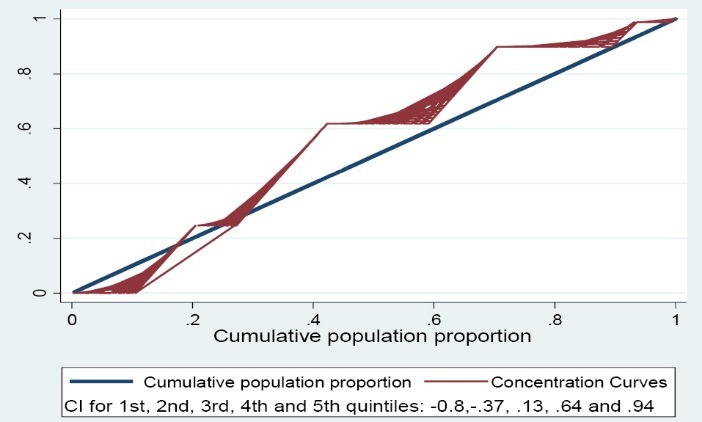
Concentration curves of economic status (x-axis) and a history of fracture (y-axis) in osteoporotic women. The line is exactly 45° show the equity line, and other curves show concentration curves [CI stands for Concentration Index].

As illustrated in Figure 2 (y-axis shows cumulative % age of ORBFs and x-axis shows the cumulative population proportion), the majority of CCs placed above the line of equality and in the range of negative values, showing that the ORBFs are more prevalent among the poorer women. It was found that 25% out of all the ORBFs are happened among 20% of women with low economic status.

## Discussion

The purpose of this study was to determine the economic inequalities caused by ORBFs among women with osteoporosis referred to the only rheumatology clinic in Sanandaj, Iran.

To the best of our knowledge, the number of studies on the decomposition of economic inequality among osteoporotic patients is few throughout the world and Iran, as well. In the current study, it was attempted, for the first time, to explain the osteoporosis-related economic inequality in Sanandaj city, applying the CI decomposition approach. Conducting such studies may help us in finding out the root causes of economic inequalities in osteoporosis among societies, which may provide vital evidences for health policy makers while their decision-makings.

Our results indicated that the economic status may play an important role in the happening of osteoporotic fractures among women; despite the higher incidence rate of osteoporosis among wealthy women, osteoporotic fractures were, remarkably, more prevalent among women with lower economic status (the ORs in the second, third, fourth and fifth quintiles were 2.45, 0.70, 0.25 and 0.06, respectively). The negative value of the CI indicated that the ORBFs are, disproportionately, concentrated among women with lower economic status (CIs in the first and the second quintiles were -0.80 and -0.37, respectively). Such a result may be common as many people with low economic status may have a lack of financial ability to procure medicines and food items. Moreover, most of these people have a lower literacy than those with higher financial ability. So, they may not have enough knowledge regarding the osteoporotic fracture preventive actions.^16^

This finding is similar with those found by Shehadeh-Sheeny et al,^17^ who reported that the lower income may be a barrier to adhere with osteoporotic medication. In their study, lower income was the most significant barrier among all the barriers. Therefore, policy makers should consider medication expenditure as an important barrier for appropriate treatments, which calls for more attention of health practitioners.

There are similar studies within which the focus is, only, on the magnitude of inequality in the ORBFs, and no one has studied the issue applying the CI decomposition approach. Okumus et al, reported that despite the moderate awareness of postmenopausal women about osteoporosis, their knowledge regarding osteoporosis risk factors and complications was poor. They concluded that having a better understanding of osteoporosis related health beliefs among women at risk may be influential, as it may play a significant role in influencing an individual’s osteoporosis preventive behaviors.^18^

Similar with those found in the present study, del Rio Barquero et al,^19^ in a study investigating bone mineral density (BMD) in two different socio-economic population groups found that the most of older people from the low economic group crossed the fracture threshold earlier than those from the high economic group. Moreover, in another study,^20^ there was a significant association between absolute poverty and age-adjusted prevalence of femoral neck osteoporosis and lumbar osteoporosis, which confirms the findings of the present study. On the other hand, Syddall et al,^21^ in England, found no relationship between economic indicators and broken bone rate. They suggested that fracture and osteoporosis may not have a strong direct social gradient. As a reason for this dissimilarity, it can be stated that their study was focused on searching for direct factors and, therefore, the indirect factors like the level of income was neglected.

The decomposition method helps to quantify the contributions of the determinants to economic inequality in health.^13^ In this study, the economic status accounted for 95.7% of inequality in the ORBFs and furthermore, the other determinants including illiteracy and unemployment accounted for the remaining 4.4%. It is noteworthy that these socio-economic factors were strongly associated with the economic status. Such results showed the usefulness of decomposition approach, as it combines the monitoring of inequality and understanding its determinants.

Brennan et al,^22^ recently, conducted a systematic review on the associations between socio-economic status (income, education, occupation, and type of residence) and BMD as well as osteoporotic fracture among adults. They concluded the limited existence of good quality evidence for associations between social inequalities in BMD and fractures occurrence. Clarification of whether the ORBFs are socially patterned may inform health care policy makers to consider the issue from a new perspective and, also, may warrant the health care providers to consider the importance of such socio-economic characteristics while planning for health education and promotion programs.

Although, as a result of the differences in the designations of the studies, the straight comparison between our findings and those found in the previous studies is difficult, it seems that the present findings are in line with the international literature within which the ORBFs are unequally distributed among societies. For instances, Zingmond et al^23^ conducting an ecological study and Leslie et al^24^ applying a case-control design concluded that the lower levels of income were associated with the higher rates of hip fracture among American and Canadian people, respectively. In another study, Johnell et al^25^ found an association between the lower levels of economic prosperity and the lower rates of hip fracture. Wang and Dixon^26^ and Piao et al^16^ conducted cross-sectional studies on women in the United States and Italy, respectively, and both concluded that the lower levels of education or income were associated with the lower levels of DXA bone mineral density.

In current decades, CID has been considered as an approach to help policymakers in defining the main issues for their interventions to reduce socio-economic inequality in health. In the present study, there were positive contributions between age, marital and educational status with inequality. Considering the remarkable role of socio-economic inequality in the unequal distribution of bone fractures among Iranian women with osteoporosis, there is an urgent need for appropriate strategies aiming to address the determinants of osteoporosis-related preventive behaviors among the poor to decrease the prevalence of consequent fractures among underprivileged women. Also, as suggested by Morasae et al, policymakers and stakeholders should bring human health to the forefront of every other issue while designing their programs.^13^ They suggested several strategies such as identifying the demographic and economic characteristics of vulnerable and disadvantaged groups and targeting the subsidy plans from the rich to the poor.

## Limitations

In the present study, family monthly income was the only indicator for economic status of the respondents due to some limitations existed while data collection. The findings in the present paper are parts of a comprehensive study with a high number of variables. As the number of items in the original questionnaire was high, the researchers decided not to include the assets/consumptions indices and, instead, to be satisfied of the family monthly income as the only indicator to measure economic status of the respondents. For future research, it is suggested to use proxies like consumption or assets indices to measure economic status of the osteoporotic patients. Moreover, since the data collection conducted by face to face interview, there may be a risk for misclassification bias. Also, because of the nature of interview, there may be a risk for underreporting by the patients. For future research, it is suggested to calculate unbiased estimation by adjusted methods for misclassification (i.e. probability bias analysis). On the other hand, due to differential misclassification, the bias-adjusted ORs cannot be determined. As the most of osteoporotic patients refer to the abovementioned osteoporosis clinic, the generalizability of the findings sounds to be established.

Moreover, due to the lack of a control group, there may be a potential for bias. It is suggested for future studies to consider the most important confounding factors such as differences in indices of bone density. In general, it is recommended that the sample size for health inequality studies to be larger, but in the present study it was not possible due to the low number of osteoporotic patients referred to the rheumatology clinic in Sanandaj and time limitations, as well.

## Conclusion

As the economic status play an important role in osteoporotic fractures occurrence among the poor women, a reorientation of women health care services in Iran focusing on underprivileged postmenopausal women seems to be necessary.

## Implications for policy and practice

Health policymakers and stakeholders should try to bridge the gap between the health promotion of the poor and the rich women with osteoporosis. Such a gap may results from inequalities in the social determinants of the disease. In order to address the prerequisites for this gap, there is an urgent need for inter-sectoral coalition between the policymakers of the health system and those of other organizations to reduce the socio-economic inequalities among osteoporotic patients.

## Acknowledgments

This research was supported by Islamic Azad University-Sanandaj Branch and Arak University of Medical Sciences. The authors thank all those patients participated in the study.

## Ethical approval

Ethical approval was provided by Ethics Committee in Islamic Azad University, Sanandaj Branch. Informed consent form was completed and signed by all the respondents.

## Competing interests

The authors have declared no conflict of interest.

## Authors’ contributions

RM was involved in the conception of the study, performed the analyses and drafted the manuscript. NM and MHK were involved in the conception of the study, interpreted the results from the analyses, and HN assisted in drafting and revising the manuscript.
